# Dynamics of thoracic endometriosis in the pleural cavity

**DOI:** 10.1371/journal.pone.0268299

**Published:** 2022-05-11

**Authors:** Takahiro Ochi, Masatoshi Kurihara, Kenji Tsuboshima, Yuto Nonaka, Toshio Kumasaka

**Affiliations:** 1 Pneumothorax Research Center and Division of Thoracic Surgery, Nissan Tamagawa Hospital, Setagaya-ku, Tokyo, Japan; 2 Department of Pathology, Japanese Red Cross Medical Center, Shibuya-ku, Tokyo, Japan; CHU Clermont-Ferrand, FRANCE

## Abstract

**Background:**

Thoracic endometriosis-related pneumothorax is a secondary spontaneous pneumothorax caused by thoracic endometriosis. Diaphragmatic endometriosis is well-studied, but visceral and/or parietal pleural lesions are not. Although surgery is an effective treatment, postoperative recurrence rates are unsatisfactory probably due to inadequate understanding of underlying pathophysiology. We aimed to clarify the clinicopathological features of thoracic endometriosis.

**Methods:**

In total, 160 patients who underwent thoracoscopic surgery from a single institution with histopathologically proven thoracic endometriosis from January 2015 to December 2019 were included. Clinicopathological characteristics and surgical outcomes were assessed retrospectively.

**Results:**

The cohort median age was 41 (range 22–53) years. Pneumothorax was right-sided in 159 (99.4%) and left-sided in only 1 (0.6%) case. Visceral and parietal pleural lesions were diagnosed in 79 (49.4%) and 71 (44.4%) patients, respectively. In total, 104 visceral pleural lesions and 101 parietal pleural lesions were detected. The S^4^ region and the dorsal 6^th^ intercostal space contained the largest number of visceral pleural (66 lesions) and parietal pleural lesions (25 lesions), respectively. Histopathological evaluation revealed endometriotic tissues, existing in the outer external elastic layer in all lesions, were localized or invaded deeply. The median follow-up period was 370 (range, 6–1824) days. The Kaplan-Meier method revealed that the 1- and 2-year postoperative recurrence rates were 13.8% and 19.3%, respectively.

**Conclusions:**

Visceral pleural endometriotic lesions may be disseminated from the visceral pleural surface and infiltrate into the pleura. Intraoperatively, careful observation of the specific sites, such as the visceral pleura of S^4^ and the parietal pleura of 6^th^ intercostal space, is important to reduce postoperative recurrence.

## Introduction

Catamenial pneumothorax (CP) is defined as recurrent secondary spontaneous pneumothorax, occurring during menstruation in reproductive-aged women. It was reported that the incidence of endometriosis-related pneumothorax occurring in the intermenstrual period, ranges from 38% to 63%, owing to endometriotic lesions in both the visceral and parietal pleura as well as the diaphragm [1, 2]. By its strict definition, CP does not include pneumothorax caused by thoracic endometriosis in the intermenstrual periods and includes primary spontaneous pneumothorax occurring in the menstrual period. Therefore, some studies had used the term thoracic endometriosis-related pneumothorax (TERP) to describe the essential pathophysiology of this disease [2–4]. In general, endometriosis affecting the diaphragm is thought to be related to the development of TERP [5–9]. However, several studies have reported that TERP patients could also present with visceral and/or parietal pleural endometriotic lesions [1, 10–12]. Diaphragmatic lesions could cause pneumothorax through the diaphragm passage [5, 11, 13] and visceral pleural lesions could cause pneumothorax through the pleural passage [6, 14]. Parietal pleura lesions, on the other hand, may not be directly related to pneumothorax, but possibly disseminate in the thoracic cavity, such as the visceral pleura. Although surgery was regarded as effective treatment for TERP, postoperative recurrence rates have remained high [1, 9–11, 15–18]. This is probably because we have not adequately understood the essential pathophysiology of thoracic endometriosis, including diaphragmatic, visceral pleural, and parietal pleural lesions.

Therefore, we aimed to clarify the clinicopathological features of thoracic endometriosis and the dynamics of the endometriotic tissues in the pleural cavity from our cases of TERP.

## Material and methods

The ethical committee at Nissan Tamagawa Hospital approved this retrospective study (No. 2019–032), and the need for informed consent from each individual was waived with a choice to opt out. In our hospital, 435 female patients aged 55 and younger underwent video assisted thoracoscopic surgery under one-lung ventilation in the lateral decubitus position for various types of pneumothoraxes from January 2015 to December 2019. A total of 166 patients, who were suspected of having thoracic endometriosis after pleural cavity inspection, underwent video assisted thoracoscopic surgery for resection of thoracic endometriosis. Among these, 160 patients were histopathologically diagnosed with thoracic endometriosis post-procedure. The following clinicopathological features were assessed by retrospective chart reviews: age at pulmonary surgery, laterality of pneumothorax, history of pelvic endometriosis, smoking history, and body mass index. History of pelvic endometriosis was confirmed by interview, transvaginal sonography, and pelvic magnetic resonance imaging.

**Fig 1:**
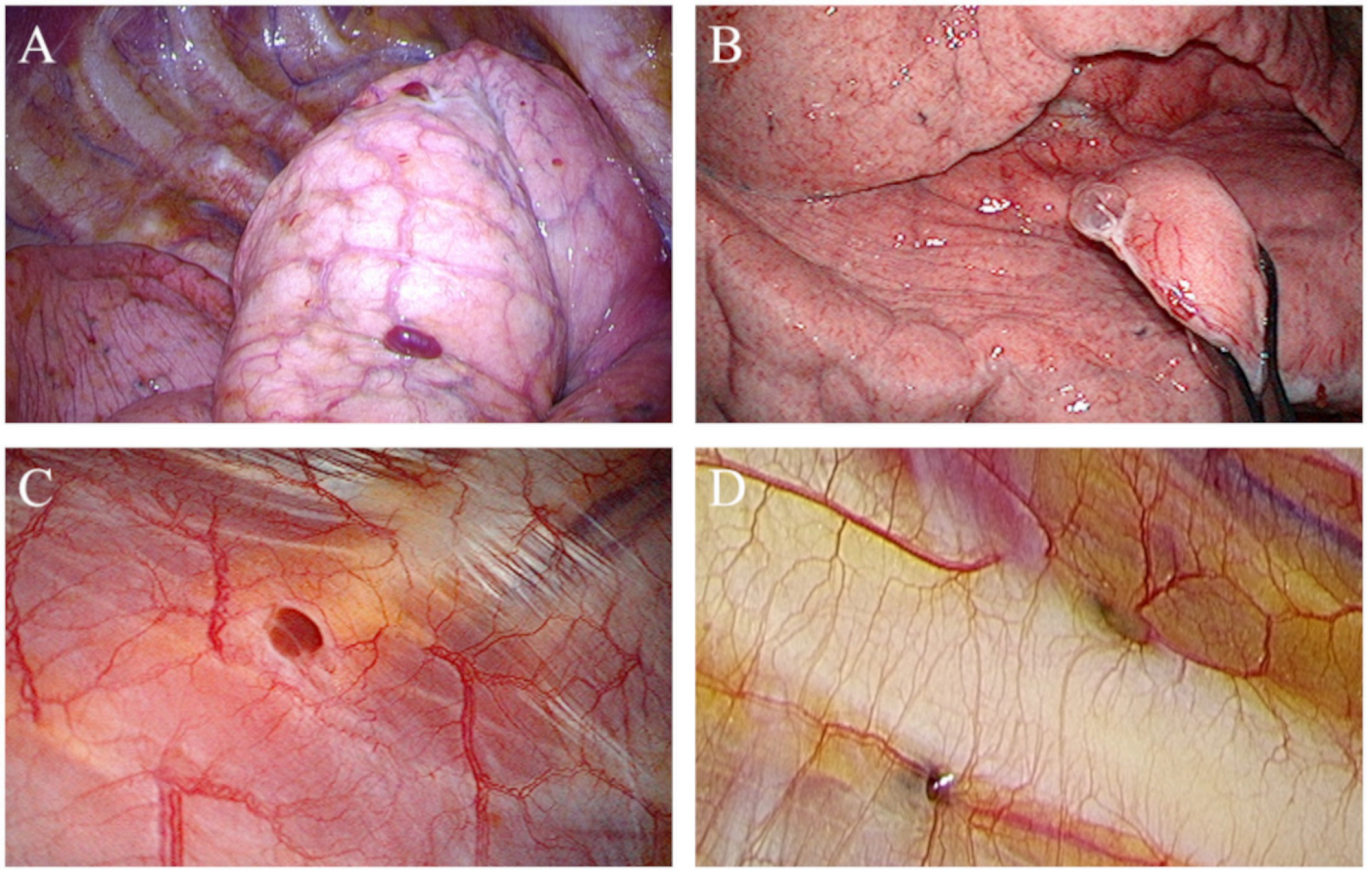
Visual aspect of visceral and parietal pleural endometriosis. Visceral pleural endometriosis showed (A) brown nodule, and (B) cystic lesion. Brown nodule was like a hematoma, to say it in another way blueberry spot. Parietal pleural endometriosis showed (C) brown nodule, and (D) dent of the pleura called depressed lesion.

In the surgical procedure, the visceral and parietal pleura, in addition to the diaphragm, were inspected carefully to identify signs of thoracic endometriosis. The diaphragm was partially resected by the stapling method and repaired by the hand suture method when diaphragmatic endometriosis was suspected. Cystic lesions in the visceral pleura, as well as the nodule-like hematomas called blueberry spots, were removed by stapling or after ligation (Fig 1A and 1B). Red or brown nodules and dents of the pleura called depressed lesions in the parietal pleura, including the intercostal muscle, were removed by partial pleurectomy (Fig 1C and 1D). All lesions were resected with careful attention to the resection margin. The areas which were partially resected owing to suspicion of thoracic endometriosis were reinforced with oxidized regenerated cellulose (ORC) in order to prevent the recurrence of pneumothorax and pleural adhesion.

All surgical specimens were assessed by hematoxylin-eosin staining. Moreover, immuno-histological examinations were performed using antibodies against estrogen receptor (ER), progesterone receptor (PgR), and CD10. All endometriotic lesions were diagnosed by confirming the presence of the endometriotic stromal or gland cells in the surgical specimens via pathological examination. Particularly with regards to endometriotic stromal cells, positive immunostaining of ER and PgR as well as CD10 could lead to a diagnosis.

In addition, we focused on visceral pleural lesions to research the mechanism of pneumothorax in visceral pleura-associated endometriosis in detail. The structure of the visceral pleura is composed of five layers: mesothelial cells, sub-mesothelial, external elastic, subpleural, and internal elastic. We microscopically evaluated the invasion level of endometriotic tissues in the visceral pleura using Elastica Van Gieson staining. All histopathological evaluations were performed by a pathologist.

Recurrence was defined by collapse of the lung, after confirming the complete expansion of the ipsilateral side postoperatively by chest radiography or computed tomography. Postoperative symptoms relating to thoracic endometriosis, such as pain, were not included in this definition as the most common reason that the patients enrolled in this study had received surgery was not such symptoms but pneumothorax. Reoperation with positive findings of thoracic endometriosis was also not included, as patients with pneumothorax recurrence did not always undergo reoperation. The Kaplan-Meier method was used to assess the postoperative recurrence rate, utilizing the statistical software program “R” Ver 3.3.2 (R Foundation for Statistical Computing, Vienna, Austria).

## Results

### Patient characteristics

The patient characteristics and clinical background of the enrolled 160 patients were described in Table 1. All patients were non-menopausal females. Most cases had right-sided pneumothorax with only one case having a left-sided pneumothorax. Fourteen patients were histologically and surgically diagnosed with pelvic endometriosis and 71 were suspected to have pelvic endometriosis by transvaginal sonography and/or magnetic resonance imaging.

**Table 1:** Patient characteristics (n = 160).

Age, years: median (range)	41 (22–53)
Laterality, n (%)	
Right	159 (99.4)
Left	1 (0.6)
Pelvic endometriosis, n (%)	
Diagnosed histologically	14 (8.8)
Suspected by imaging study	71 (44.4)
Smoking history, yes, n (%)	49 (30.6)
Body mass index: mean±SD	19.8±2.2

SD, standard deviation.

### Distribution of endometriotic lesions in the pleural cavity

**Fig 2:**
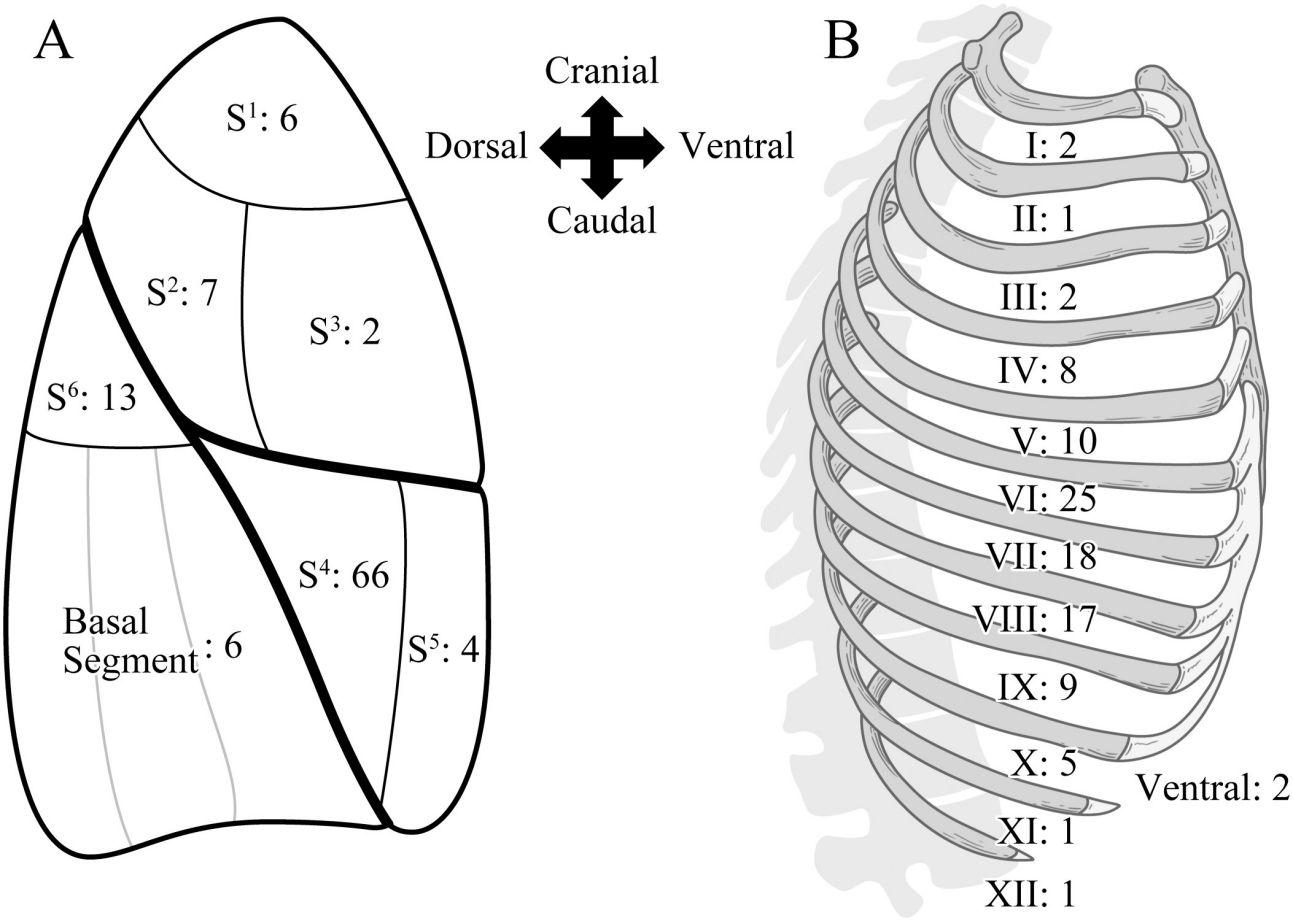
Distribution of endometriotic lesions. (A) Visceral pleural lesions, and (B) Parietal pleural lesions. Eighty-six visceral pleural lesions (82.7%) out of 104 were observed in S^4^, S^6^, and S^2^. Eighty-seven parietal pleural lesions (86.1%) out of 101 were observed in 4^th^-9^th^ dorsal intercostal space.

All the 160 patients had diaphragmatic endometriotic lesions. Endometriosis in the visceral pleura was diagnosed in 79 patients (49.4%), and endometriosis in the parietal pleura in 71 patients (44.4%). Thirty-six patients had both the visceral and parietal endometriotic lesions. Multiple lesions were confirmed in some patients in both the visceral and parietal pleura. Regarding visceral pleural lesions, one lesion was confirmed in 61 patients, two lesions were in 11, and three lesions were in 7. These added up to 104 lesions in total. Regarding parietal pleural lesions, one lesion was confirmed in 51 patients, two lesions were in 12, three lesions were in 6, and four lesions were in 2. These added up to 101 lesions in total. Visceral pleural endometriosis showed more cystic lesions rather than blueberry spots. The number of positive cases for cystic lesions, blueberry spots, and combined cases were 80 (76.9%), 14 (13.5%), and 10 (9.6%), respectively. The distribution of visceral and parietal pleural lesions under thoracoscopy was shown in Fig 2. For visceral pleural lesions, 66 lesions (63.5%) were detected in S^4^, 13 lesions (12.5%) in S^6^, and 7 lesions (6.7%) in S^2^, respectively. Eighty-six lesions (82.7%) were found concentrated in the area where the upper, middle, and lower lobes of the lung intersect. Among these, eighty-two lesions (82/86: 95.3%) existed within the interlobar surface or at the border of each segment. In the parietal pleural lesions, 25 lesions (24.8%) were detected in the dorsal 6^th^ intercostal space (ICS). Eighty-seven lesions (86.1%) were found in the dorsal 4^th^-9^th^ ICS.

### Pathological features of endometriotic tissues

Endometriotic stromal cells were confirmed in all 160 (160/160: 100%) diaphragmatic lesions, and endometriotic gland cells were found in 61 (61/160: 38%). In 104 visceral pleural lesions, endometriotic stromal cells were confirmed in all lesions (104/104: 100%). In contrast, endometriotic gland cells were confirmed in only three (3/104: 2.9%). In 101 parietal pleural lesions, endometriotic stromal cells were confirmed in 100 lesions (100/101: 99%) and endometriotic gland cells were confirmed in 17 lesions (17/101: 17%). All endometriotic stromal tissues were stained positive for ER, PgR, and CD10, and all endometriotic gland tissues were stained positive for ER and PgR, but negative for CD10.

**Fig 3:**
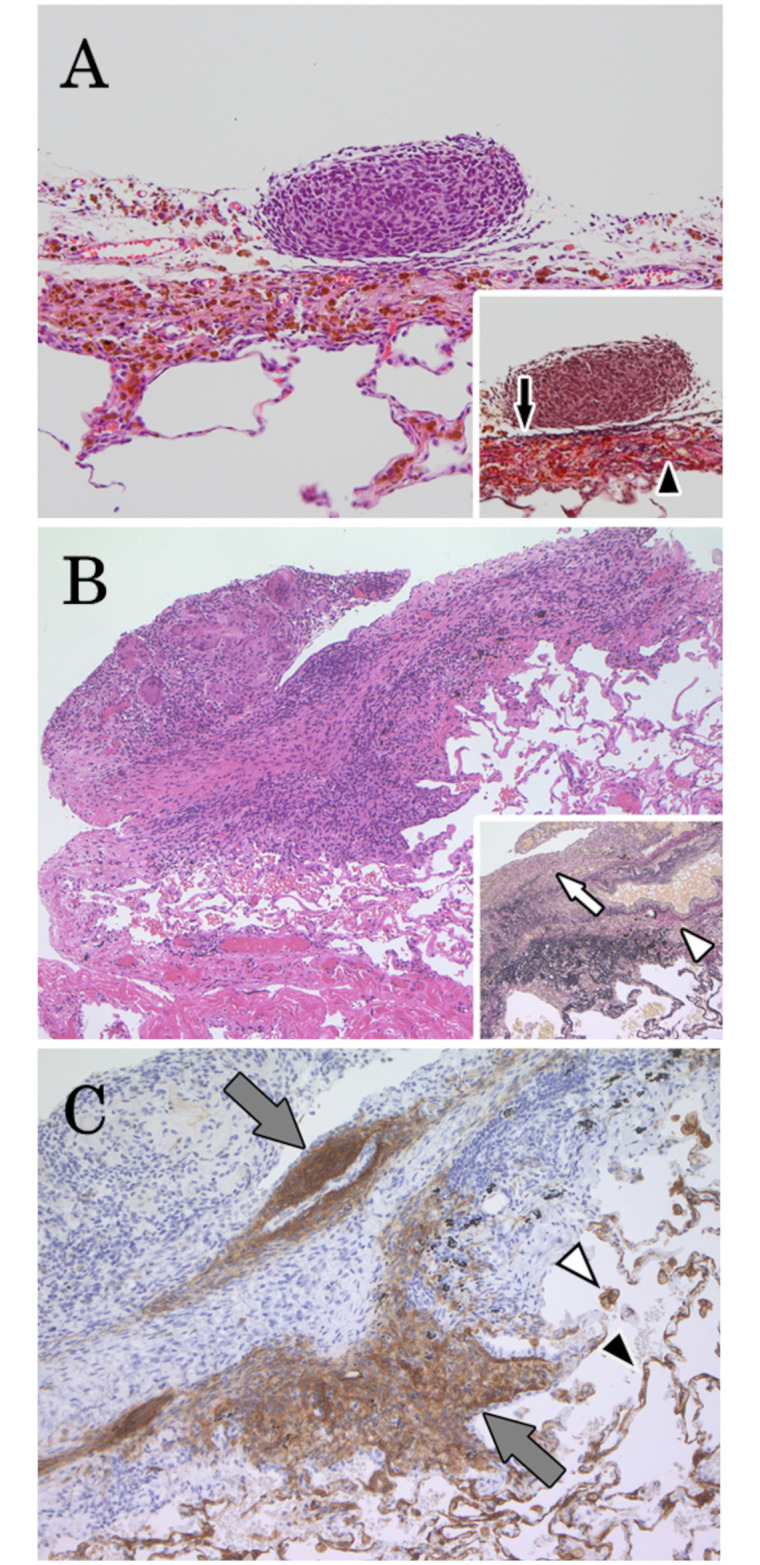
Localization of endometriotic lesions in the visceral pleura. (A) Endometriotic tissue adhered to the surface of the visceral pleura. Note that marked hemosiderin deposition was observed in the entire pleural wall. (Hematoxylin-Eosin stain, inset: the external and internal elastic layer were indicated by black arrow and arrowhead, respectively (Elastica von Gieson stain)). (B) Endometriotic lesions surrounded by granulation and/or fibrous tissue accompanied with inflammatory cells invaded into the lung tissue through the visceral pleura. (Hematoxylin-Eosin stain, inset: white arrow and arrowhead indicated the break of the external and internal elastic layer caused by the invasion of endometriotic tissue, respectively (Elastica von Gieson stain)). (C) Endometriotic stromal cells in the endometriotic lesion were immune-positive for CD10 (gray arrows) in the same tissue of Fig 3B. Note that the anti-CD10 antibody was cross-reactive to alveolar stromal cells (black arrowhead) and alveolar macrophages (white arrowhead).

We then histopathologically investigated in greater detail, the 104 visceral pleural lesions, paying close attention to location and tissue invasive behavior. Some endometriotic tissues stayed on visceral pleural surfaces, while others demonstrated inward tissue invasion from the visceral pleural surfaces (Fig 3). The depth of invasion varied per lesion, with some reaching up to the subpleural layer or pulmonary alveolus; however, all lesions appeared to exist continuously from the outer side of the external elastic layer of the visceral pleura. Endometriotic tissues localized in the visceral pleural surface were confirmed in 54 lesions (51.9%), tissues invading the external elastic layer in 20 (19.2%), and tissues invading the internal elastic layer in 30 (28.8%) (Table 2).

**Table 2:** Invasion depth of 104 visceral pleural endometriotic lesions, n (%).

Pleural surface	54 (51.9)
External elastic layer	20 (19.2)
Internal elastic layer	30 (28.8)

### Surgical outcomes

**Fig 4:**
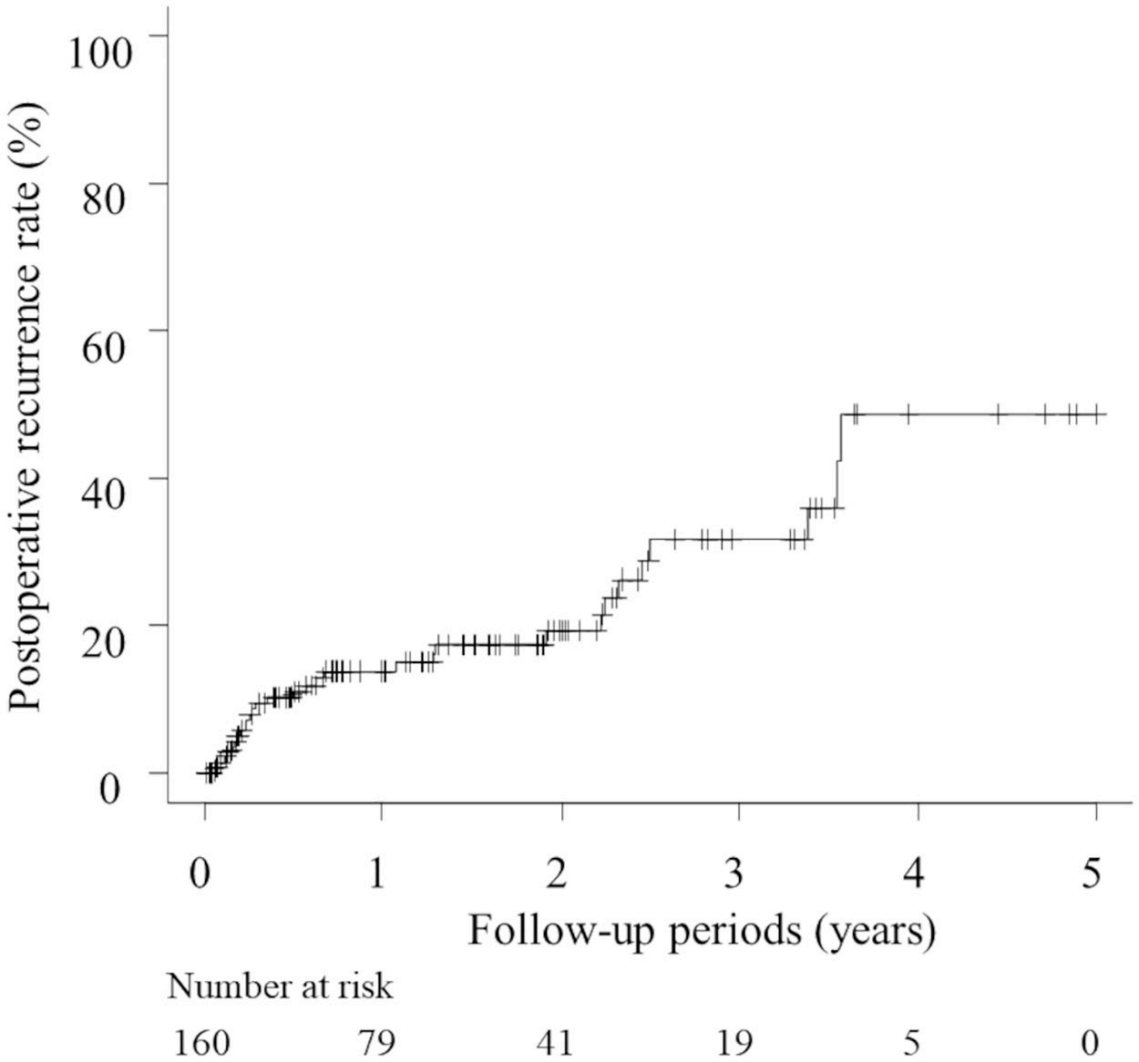
Kaplan-Meier curve of postoperative recurrence rate.

**Fig 5:**
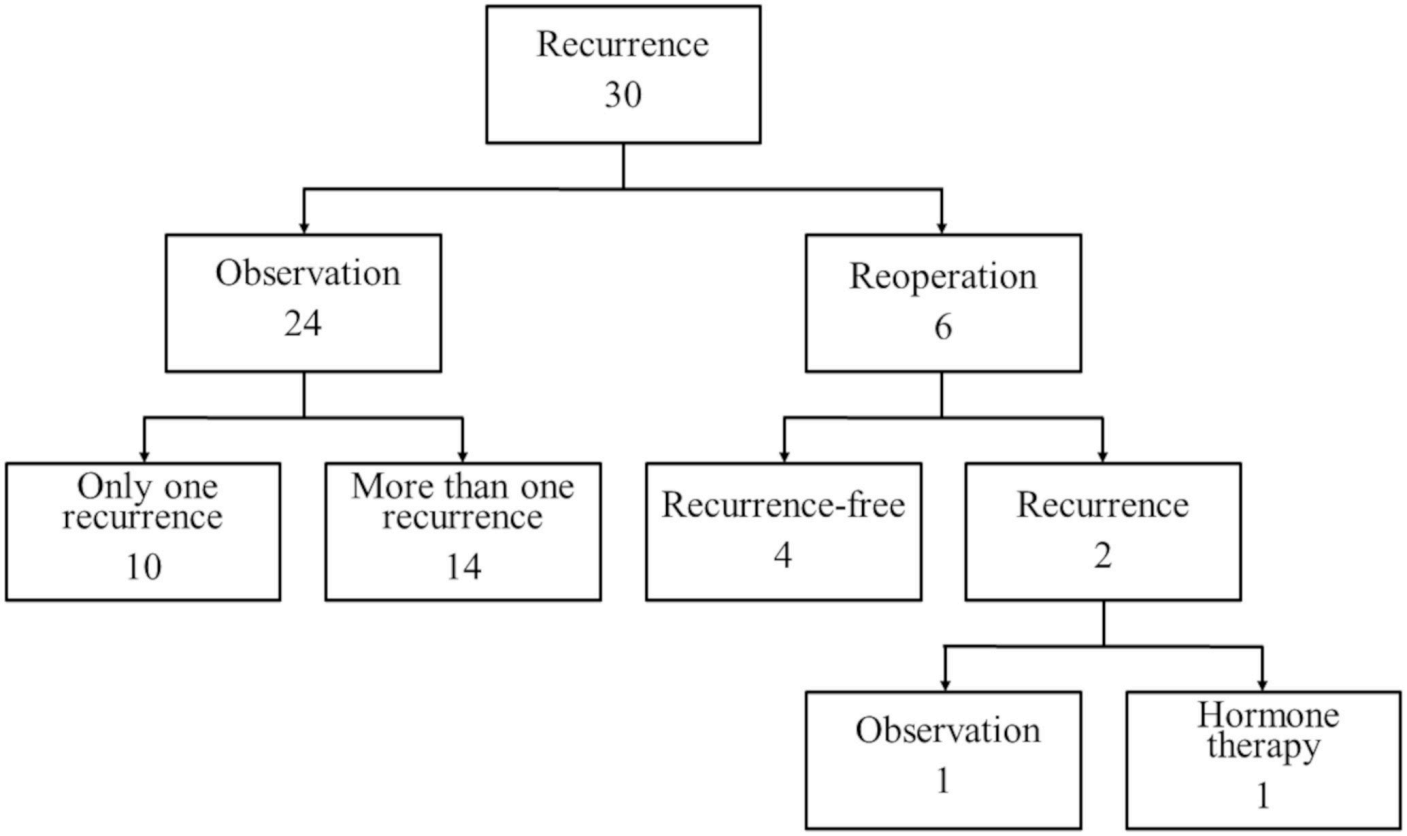
Flow chart of treatments for 30 patients with postoperative recurrences.

The median follow-up period was 370 (range 6–1824) days. A total of 30 patients (30/160: 18.8%) developed postoperative recurrences. The mean number of recurrences was 3.1±3.7. The median time to recurrence after surgery was 195 (range 23–1303) days. Twenty-two patients (22/30: 73.3%) developed recurrences within 2 years. The Kaplan-Meier method revealed that the 1- and 2-year postoperative recurrence rates were 13.8% and 19.3%, respectively (Fig 4). Of the 30 patients with postoperative recurrences, 24 received no additional treatments and were followed up: 10 had one recurrence and have not relapsed since, whilst 14 had multiple recurrences; however, postoperatively the pneumothorax developments were noted to be reduced in both frequency and intensity (Fig 5). The other 6 patients underwent reoperations because the rate of pneumothorax developments did not decrease post-surgery. The frequency of recurrences was determined by dividing the total number of onsets for all patients by their total observation period, according to the previous report [19]. Using this calculation method, the number of cumulative recurrences were 40 and 54, and the frequencies of cumulative recurrences were 4.51 and 1.53 times per year in the patients who underwent reoperation and those who did not, respectively. In those who underwent reoperation, all visceral pleural endometriotic lesions were confirmed in S^4^, S^6^, and S^2^ (5/5: 100%) and majority of the parietal pleural lesions were in the dorsal 5^th^-7^th^ ICS (3/5: 60%). In those who did not undergo reoperation, most of the visceral pleural lesions were confirmed in S^4^, and S^6^ (12/13: 92%) and majority of the parietal pleural lesions were in the dorsal 4^th^-8^th^ ICS (13/18: 72%). In addition, the detailed characteristics of the 6 patients who received reoperations were shown in Table 3. We resected any suspicious lesions and performed pleural covering of the specific sites of thoracic endometriosis with ORC for all cases. The residual endometriosis was histopathologically confirmed in 3 cases (3/6: 50%); the locations of endometriosis were diaphragm, S^1^, S^4^ in the visceral pleura, and dorsal 4^th^ ICS in the parietal pleura. Of the 6 patients who underwent reoperations, 4 patients have not relapsed. Of the 2 cases of relapse, one had 2 recurrences following reoperation and has not relapsed since, whilst the other had 1 recurrence, has started hormone therapy (Dienogest) and has not relapsed since.

**Table 3:** Detailed characteristics of the 6 patients who received reoperations.

Patient number	Age, y	Laterality	Localization of thoracic endometriosis proven histopathologically						Number of recurrences after 2^nd^ operation
			1^st^ operation			2^nd^ operation			
			D	V	P	D	V	P	
1	35	Right	yes	S^4^, S^6^	none	none	S^1^	Dorsal 4^th^ ICS	0
2	36	Right	yes	none	none	none	none	none	0
3	37	Right	yes	S^6^	none	yes	none	none	0
4	40	Right	yes	none	none	none	S^4^	none	0
5	28	Right	yes	S^2^, S^4^	Dorsal 1^st^, 5^th^-7^th^ ICS	none	none	none	2^†^
6	47	Right	yes	none	Ventral	none	none	none	1^‡^

^†^This patient had two recurrences and has not relapsed since.

^‡^This patient had one recurrence; following the administration of Dienogest, has not relapsed since.

D: diaphragm; V: visceral pleura; P: Parietal pleura; ICS: intercostal space.

## Discussion

TERP is caused by the air entering through two different passages: the trans-diaphragmatic passage and trans-pleural passage [5, 6, 13, 14]. It is hypothesized that, in the former, air flows into the peritoneal cavity through the fallopian tube, reaches the diaphragm alongside the clockwise rotation of peritoneal fluids and enters the intrathoracic space through a diaphragm defect that developed secondary to endometriotic tissue invasion-related injury [5, 11, 13, 20]. The pressure difference between the pleural and abdominal cavities might facilitate the inflow of air into the pleural cavity as the pleural cavity is kept under negative pressure, while the abdominal cavity is kept under positive pressure. Moreover, regarding the latter, it is hypothesized that air enters the intrathoracic space through a lung surface defect caused by the deciduation of endometriotic lesions in the visceral pleura during menstruation [6, 14]. Fukuoka et al. has reported on the possibility that TERP is caused by the trans-pleural passage or by the trans-diaphragm passage, which develops pneumothorax in the intermenstrual period [2]. Endometriosis in the parietal pleura could be disseminated from diaphragmatic endometriosis owing to flow effects from pleural effusion and afterward, possibly disseminates within the pleural cavity. Thus, parietal pleural endometriosis may not be a direct cause of pneumothorax, but it possibly increases the risk for thoracic endometriosis and plays an important role in the development of TERP.

**Fig 6:**
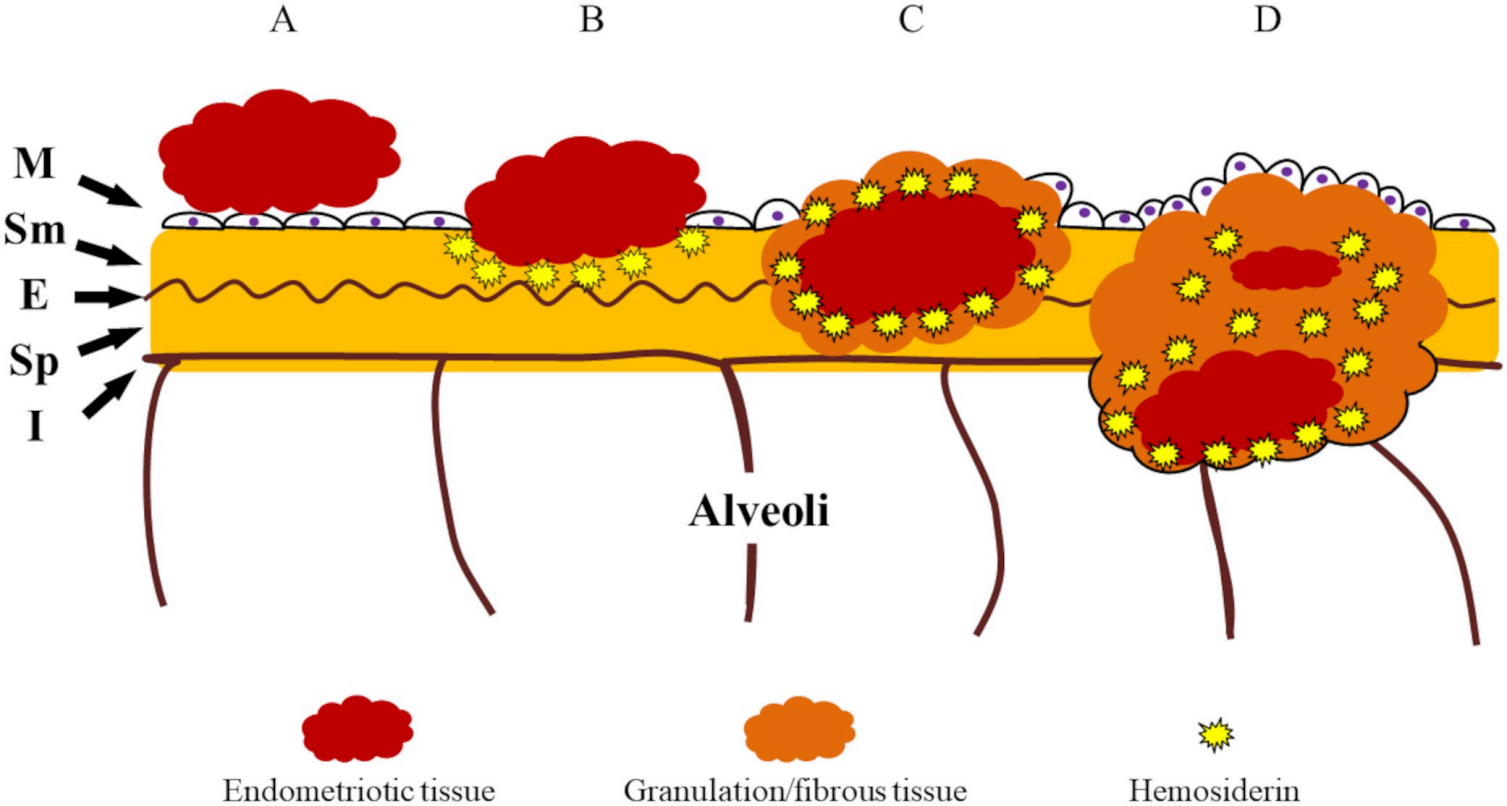
Schematic illustration of breaking of the visceral pleural structure in endometriosis. Endometriotic tissue migrating in the thoracic space may be located on the surface of the visceral pleura (A). Subsequently, proliferation and breakdown of the endometriotic tissue may occur during menstruation cycles on the surface of pleura as the same fashion of uterus. It may lead to bleeding, tissue repair, and inflammation of the endometriotic lesion involving the surrounding pleural tissue, suggesting stepwise breaking of the pleural structure (mesothelial, sub-mesothelial, external elastic, subpleural, and internal elastic layer) (B-D).

The pathogenesis of thoracic endometriosis has been mainly explained as follows: (i) migration of pelvic endometriosis to the diaphragm [4], (ii) lung metastasis of endometriosis through blood vessels [21], and (iii) coelomic metaplasia of the epithelium in the thoracic cavity [22]. The present study revealed that disseminated endometriotic tissues invade the visceral pleura, which could support the theory that pelvic endometriosis may migrate to the diaphragm. Diaphragmatic endometriotic tissues were assumed to disseminate to the visceral and/or parietal pleura, owing to flow effects from pleural effusion. As previous research has suggested that endometriotic tissues could relate to the inflammatory cytokine production [23], we assumed that endometriotic tissues adhered to the visceral pleural surface could result in inflammation of the surrounding tissues. Proliferation and breakdown of the endometriotic tissue may occur during menstruation cycles on the surface of the visceral pleura as the same fashion of uterus, which could lead to bleeding, tissue repair, and inflammation of the endometriotic lesion involving the surrounding pleural tissue, suggesting stepwise breaking of the pleural structure (mesothelial, sub-mesothelial, external elastic, subpleural, and internal elastic layer) (Fig 6).

In this study, 86 visceral pleural lesions (82.7%) were detected in the S^4^, S^6^, and S^2^ regions and 87 parietal pleural lesions (86.1%) were detected within the 4^th^-9^th^ dorsal ICS. This begs the question as to why endometriotic tissues tend to stay and engraft in these limited sites. Endometriotic tissues are benign, but tend to disseminate with comparative ease. Although malignant tissues are easily able to disseminate at any site, endometriotic tissues need a stable and quiet environment to engraft and self-proliferate. Berlanda N et al. reported that pelvic endometriosis tends to be engrafted on stable areas where the organs were less likely to adversely affect growth [24]. This could be termed the “shelter effect.” It is assumed that the specific sites in the pleural cavity are likely to be stable and quiet spaces not affected by heartbeat and respiratory movement. We theorize that such ideal environments are present within limited sites in the margin of S^4^, S^6^, and S^2^ in the visceral pleura and within a limited place in the 4^th^-9^th^ dorsal ICS.

There are several reports about the treatment for TERP. Previous studies reported that recurrence rates in patients undergoing surgery ranges from 8% to 40% [1, 9–11, 15–18]. According to Alifano et al., 114 patients underwent pulmonary surgery including resection of not only the diaphragm but also the visceral and parietal pleura with 29 of these patients later proven to have endometriosis histopathologically. In a subsequent 33-month follow-up period, the postoperative recurrence rate of TERP patients in whom pneumothorax occurred during menstruation was 32% and that in whom pneumothorax occurred during their intermenstrual periods was 27% [1]. Marshall et al. studied eight patients who underwent surgery including diaphragmatic resection, bullectomy, pleurodesis, and pleurectomy. Six of these patients received hormonal pharmacotherapy and postoperative recurrence rate was 37.5%, during follow-up ranging 27 to 63 months (mean 48 months) [17]. Joseph et al. reported that the 1-year recurrence rate in 21 TERP patients who underwent surgical pleurodesis was 30% compared to 60% in patients who received hormone therapy [9]. Moreover, relatively strong adverse effects [25] and recurrence risk after hormone therapy [26] have also been reported. Thus, hormone therapy for thoracic endometriosis is not conducted generally in our institution. Resection of visceral and parietal pleural lesions is performed in addition to diaphragm resection via a surgical procedure. Moreover, we reinforced with ORC, the resected areas alongside the specific sites for thoracic endometriosis, including not only the diaphragm but also the visceral and parietal pleura, considering the possibility that minute endometriotic lesions could be unrecognized. This surgical procedure was based on reports stating that reinforcement with ORC could influence the development of visceral pleura thickening and may reduce postoperative recurrence in spontaneous pneumothorax patients [27–29]. Using our therapeutic strategy, the 1- and 2-year postoperative recurrence rates in TERP patients were 13.8% and 19.3%, respectively. The number of patients enrolled in previous studies was smaller than that in the present study. Furthermore, postoperative recurrence rates were assessed using simple recurrence rate ratios with no link to follow-up periods. This method does not take the time period to recurrence into consideration. In contrast, our study utilized the Kaplan-Meier method, which specifically measures event probability within specific time intervals. Thus, previous results are not necessarily comparable to ours owing to our more exacting methods of analysis, although the present research showed that the surgical outcomes in our institution were acceptable. Regarding visceral pleural endometriotic lesions, one should pay attention to their appearance when inspecting the thoracic cavity. It is widely known that endometriosis in the visceral pleura can exhibit a blueberry spot appearance. However, our study revealed a greater prevalence of small cystic lesions rather than blueberry spots. The exact pathophysiology of cystic lesions remains uncertain, but there is a possibility that growth of endometriotic tissues causes terminal bronchiole stenosis, resulting in the creation of a check-valve mechanism or destroys and invades environmental tissues. Because rupture of cystic lesions causes TERP, careful inspection of the visceral pleura is important in order to avoid overlooking small cystic lesions.

Histopathological diagnosis revealed that endometriotic stromal cells were confirmed in all lesions, but the positivity ratios of endometriotic gland cells were relatively low in diaphragm, visceral, and parietal pleural lesions. Haga et al. showed that endometriotic stromal cells were detected in all specimens of the resected diaphragm, but endometriotic gland cells were detected in only 25% [30]. These results would suggest endometriotic gland cells are not always present in endometriotic lesions. Confirming endometriotic stromal cells in a specimen using immunostaining of ER, PgR, and CD10 is important to diagnose thoracic endometriosis. However, this might occur if the endometriotic lesions could be damaged during the biopsy and/or histological analysis processes. The average number of sections per lesion in the visceral pleura was 1.19±0.62 in this study. Considering the recent study that 24 TERP cases (24/26: 92%) showed endometriotic glands [31], it is possible that the relatively small number of sections per lesion could be related to the low positive rate of the endometriotic glands.

Apart from this, the present study has three limitations. First, this was a single institutional retrospective study. In general, a larger multi-center comparative cohort study would be essential to demonstrate precise pathophysiology. However, this would also be very suitable to treat and research a larger sample of patients suffering from TERP, using a standardized treatment policy of a single facility specializing in pneumothorax, because TERP is a rare disease and lesions may be too minute for researchers unfamiliar with this disease to detect easily. Second, there might be overlooked or invisible endometriotic lesions, even though we inspected the visceral and parietal pleura in detail, including the interlobar, mediastinal areas, lung basal surface, and so on. In our institution, almost all patients who were suspected to have TERP received preoperative intrathoracic examinations under local anesthesia in the dorsal and lateral decubitus position using flexible thoracoscopes, because we assumed that increase of opportunity to observe in the pleural cavity could reduce the number of minute lesions being overlooked. Nevertheless, half of the patients who underwent reoperations after their 1st surgery for TERP were found to have residual thoracic endometriosis. Finally, we argued the specific sites for thoracic endometriosis in this study, however it cannot be denied that endometriotic lesions are present throughout the thoracic cavity and they just get conspicuous in those areas. Hwang et al. stated that thoracic endometriosis was mainly distributed on the right upper lobe (9/15: 60%) [32]. This difference in distribution trends might be a result of missing lesions which are difficult to recognize. Other inspection methods and/or treatments, as well as progressing thoracoscopy technology, will be required to elucidate the precise pathophysiology of TERP and further decrease postoperative recurrence.

## Conclusions

The visceral pleural lesions in TERP could mainly develop from pleural dissemination and infiltration into the lung. It may be essential to make careful observation of the following specific sites for thoracic endometriosis: visceral pleura in S^4^, S^6^, S^2^, and parietal pleura in dorsal ICS, because visceral and parietal pleural endometriosis, in addition to diaphragm endometriosis, could participate in postoperative recurrence of TERP.

## Supporting information

S1 File Data set of patients and lesions. (XLSX)
